# Strain‐Specific Safety Evaluation of *Akkermansia muciniphila* Akk11: Comprehensive Genotypic, Phenotypic, and Toxicological Assessment

**DOI:** 10.1002/fsn3.71154

**Published:** 2025-11-04

**Authors:** Xiaowen Wang, Yixuan Fan, Yao Dong, Yinan Zhang, Xinyu Tan, Zhonghui Gai, Yukun Sun, Shuguang Fang

**Affiliations:** ^1^ Wecare Probiotics R&D Centers, Wecare Probiotics Co., Ltd. Suzhou China; ^2^ Shanghai Institute of Quality Inspection and Technical Research Shanghai China

**Keywords:** *Akkermansia muciniphila* Akk11, genotypic analysis, phenotypic analysis, probiotics, strain‐specific safety, toxicology

## Abstract

*Akkermansia muciniphila* is increasingly regarded as a next‐generation probiotic with clinical and commercial potential. This study aimed to evaluate the strain‐specific safety and probiotic characteristics of Akk11, isolated from the feces of healthy infants. The genotypic profile of Akk11 was assessed via whole‐genome sequencing, including antibiotic resistance, virulence factors, and metabolic pathway annotations. Phenotypic analyses included antibiotic susceptibility, mucin degradation, biogenic amine and D‐/L‐lactic acid production, and hemolytic activity. Stress tolerance and cytotoxicity were performed using simulated gastrointestinal conditions and Caco‐2 cells. In vivo safety was further evaluated through acute oral toxicity, bacterial reverse mutation, mammalian erythrocyte micronucleus, and 90‐day sub‐chronic toxicity, following international guidelines. Akk11 showed 98.36% genomic similarity to the type strain *adeF* with no mobile genetic elements or transferable resistance. Phenotypically, Akk11 exhibited intrinsic resistance to several antibiotics while lacking harmful metabolic activities and excellent simulated gastrointestinal stress tolerance with non‐cytotoxicity. No adverse effects were observed in any of the in vivo toxicity studies, even under long‐term oral administration of bacterial powder preparations containing both viable and non‐viable cells. The NOAEL was 9 × 10^11^ AFU/kg/day, the highest dose tested in both sexes, using a lyophilized preparation that contained approximately 20%–30% non‐viable Akk11 cells (n‐AFU). Akk11 possesses a robust, strain‐specific safety profile and probiotic potential, providing a scientific basis for its application in next‐generation probiotic products.

## Graphical abstract

*Akkermansia muciniphila* Akk11 showed strain‐specific safety: 98.36% genomic similarity to the type strain, with no mobile elements, no harmful metabolites, strong simulated GI tolerance, and no Caco‐2 cytotoxicity. Acute, reverse‐mutation, micronucleus, and 90‐day studies found no adverse effects; NOAEL was 9 × 10^11^ AFU/kg/day using a lyophilized powder containing 20%–30% non‐viable cells. Findings support Akk11's robust safety and next‐generation probiotic potential.

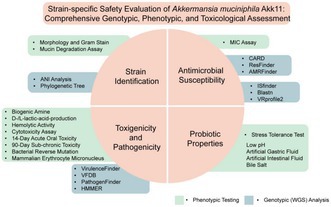

## 1 Introduction

The human gut harbors diverse and dynamic microbial communities, collectively known as the gut microbiota, which play an essential role in maintaining host health and preventing disease. These microbial populations are influenced by various factors, including diet, lifestyle, and medical interventions. Disruptions in gut microbial balance, referred to as dysbiosis, have been linked to multiple health conditions such as metabolic syndrome, neurodegenerative disorders, gastrointestinal diseases, cardiovascular diseases, and cancer (Ma et al. 2023). Probiotic interventions, aimed at modulating the gut microbiota and its metabolic activities, are increasingly recognized as a promising strategy for disease prevention and health promotion. Ensuring the safety and efficacy of probiotics is thus critical to their successful application in both clinical and commercial contexts (Ma et al. 2023).

*Akkermansia muciniphila* , a representative mucin‐degrading bacterium from the Verrucomicrobia phylum, was first isolated two decades ago and has since attracted considerable attention as a next‐generation probiotic candidate (Derrien et al. 2004). Reduced abundance of *A. muciniphila* in the gut microbiota has been associated with metabolic disorders, obesity, type 2 diabetes, hepatic steatosis, chronic inflammation, and altered responses to cancer therapies (Cani et al. 2022). Preclinical studies have demonstrated that supplementation with *A. muciniphila* can ameliorate these conditions, highlighting its potential roles in modulating host metabolism and immune responses (Cani et al. 2022; Ghaffari et al. 2023). These findings underscore the growing interest in developing *A. muciniphila* ‐based probiotic products; however, the safety profile of specific strains remains a prerequisite for their clinical and commercial application.

With the growing public interest in functional foods and microbiome‐targeted interventions, there is an increasing demand for safe, standardized probiotic products. To ensure consumer protection, international regulatory authorities such as the World Health Organization (WHO), U.S. Food and Drug Administration's Generally Recognized As Safe (FDA's GRAS), United States Pharmacopeia (USP) and European Food Safety Authority (EFSA) have established specific guidelines for probiotic safety evaluation (Araya et al. 2002; Boyte et al. 2024; Koutsoumanis, Allende, Alvarez‐Ordóñez, et al. 2020; Hazards et al. 2021). These frameworks emphasize the importance of strain‐specific assessments, focusing on characteristics such as artificial gastrointestinal stress tolerance, adhesion to intestinal epithelial cells, absence of virulence factors, antibiotic resistance profiles, and non‐toxicity. Despite these established standards, comprehensive safety data for the *A. muciniphila* strain, particularly regarding both viable and non‐viable bacterial preparations, remain limited and urgently needed.

Unlike well‐established probiotic species such as *Lactococcus*, *Lactobacillus*, *Streptococcus* and *Enterococcus* (De Melo Pereira et al. 2018), *A. muciniphila* represents a relatively novel candidate in the probiotic field. Current research has predominantly focused on the type strain *A. muciniphila* Muc^T^ (ATCC BAA‐835/DSM 22959), with pasteurized forms having been approved as a novel food by EFSA (Turck et al. 2021). However, *A. muciniphila* has not yet been granted Qualified Presumption of Safety (QPS) status, largely due to concerns regarding strain‐specific variability in antibiotic resistance, potential virulence factors, and limited toxicological data across different formulations. To address these gaps, we isolated *A. muciniphila* Akk11 from healthy infant feces and performed a comprehensive strain‐specific safety evaluation, encompassing genotypic and phenotypic characterization as well as in vitro and in vivo toxicological assessments in accordance with FDA's GRAS, USP, EFSA, and WHO/FAO guidelines. Particular attention was given to evaluating the safety of bacterial preparations containing both viable and non‐viable cells, reflecting realistic conditions in probiotic product development. This study aims to provide a robust scientific basis for future clinical trials and the potential commercialization of Akk11 as a next‐generation probiotic.

## 2 Materials and Methods

### 2.1 Strain and Culture Conditions

Strain Akk11 was isolated from healthy infant feces and obtained from Wecare Probiotics Co. Ltd. (Suzhou, China). The strain was routinely cultured under anaerobic conditions at 37°C in brain heart infusion (BHI) medium supplemented with 0.2% (w/v) mucin. Morphological features were assessed using Gram staining and scanning electron microscopy (ZEISS Sigma 300). All chemicals and reagents, including Caco‐2 cells, assay kits, and culture media, were purchased from recognized commercial suppliers and are listed in Table S1.

### 2.2 Genotypic Characterization

Genomic DNA of Akk11 was extracted and subjected to whole‐genome sequencing using both PacBio Sequel II (long‐read) and Illumina NovaSeq (short‐read) platforms. Raw data were quality controlled using fastp (Chen et al. 2018), and hybrid genome assembly was performed with Unicycler (Wick et al. 2017). Circular genome maps were generated using Circos v0.69.3 (Krzywinski et al. 2009). Average nucleotide identity (ANI) was calculated using fastANI (Jain et al. 2018), with *A. muciniphila* Muc^T^ (CP071807) as the reference. Phylogenetic analysis was conducted using the neighbor‐joining method in MEGA 7 (Hou et al. 2023). Functional annotation was performed using Gene Ontology (GO) and Kyoto Encyclopedia of Genes and Genomes (KEGG) databases (Gu et al. 2024). Antibiotic resistance genes (ARGs), virulence factors, and mobile genetic elements (MGEs) were screened against CARD (Jia et al. 2016), ResFinder (Zankari et al. 2012; Malberg Tetzschner Anna et al. 2020), AMRFinderPlus, ISfinder (Xie and Tang 2017), VRprofile2 (Wang et al. 2022), VirulenceFinder, and VFDB databases using default parameters (Chen et al. 2005). Pathogenicity potential was predicted using PathogenFinder (Cosentino et al. 2013). Biogenic amine biosynthesis genes were screened using Hidden Markov Models (HMMs) based on conserved enzyme sequences (Gardini et al. 2016; Eddy 2009). Detailed software versions and analysis parameters are provided in Table S2.

The whole‐genome sequence of Akk11 has been deposited in the NCBI repository under BioProject accession number PRJNA1202374 and will be made publicly available upon publication acceptance.

### 2.3 Phenotypic Characterization

The phenotypic properties of Akk11 were evaluated in accordance with EFSA guidelines. Morphology was assessed by Gram staining and scanning electron microscopy (SEM, ZEISS Sigma 300) after culturing in BHI medium supplemented with 0.2% (w/v) mucin under anaerobic conditions (37°C, 48 h). Mucin‐degrading activity was determined via SDS‐PAGE (12.5% polyacrylamide gel) analysis of ethanol‐precipitated mucin residues as described previously (Abe et al. 2010). Antibiotic susceptibility was assessed by determining the minimum inhibitory concentration (MIC) using the broth microdilution method against eight antibiotics: ampicillin, gentamicin, kanamycin, streptomycin, tetracycline, ciprofloxacin, colistin, and fosfomycin. MIC values were interpreted according to EFSA breakpoint guidelines (EFSA Panel on Additives and Products or Substances used in Animal Feed et al. 2018; Clinical and Laboratory Standards Institute 2025). Biogenic amines were quantified and qualified by LC–MS/MS method with some modifications (Pawar et al. 2014; Mayr and Schieberle 2012). Detailed LC–MS/MS method protocol is provided in Table S3. D‐/L‐lactic acid production was measured using a D‐/L‐Lactic Acid (Rapid) Test Kit following the manufacturer's instructions. Hemolytic activity was determined by streaking Akk11 onto Commercial Ready‐to‐use Columbia blood agar supplemented with 5% sheep blood, followed by incubation at 37°C for 48 h and visual inspection for hemolysis zones (Kawacka et al. 2022).

### 2.4 Functional Properties

The functional properties of Akk11 were evaluated to assess its gastrointestinal stress tolerance and cytotoxicity. Stress tolerance was assessed by exposing Akk11 to acidic conditions, bile salts, and simulated gastrointestinal fluids (Wu et al. 2022). For acid tolerance, Akk11 cells were resuspended in saline and inoculated into BHI agar adjusted to pH 2.5. Bile salt tolerance was determined in BHI medium containing 0.3% (w/v) bovine bile salts. Artificial gastric and intestinal fluids were prepared using 3 g/L pepsin in 0.5% saline (pH 3.5) and 0.1% trypsin in 0.5% NaCl (pH 8.0), respectively (Chen et al. 2024). Akk11 was incubated in these media for up to 3 h, and viable counts were measured by plate counting and expressed as active fluorescent units (AFU/mL).

Cytotoxicity was evaluated using Caco‐2 cells by CCK‐8 and LDH release assays (Committee et al. 2018). For the CCK‐8 assay, Akk11 fermentation broth was co‐incubated with Caco‐2 cells in 96‐well plates overnight at 37°C with 5% CO_2_. Cell viability was determined by measuring absorbance at 450 nm after CCK‐8 reagent treatment. For the LDH assay, Caco‐2 cells were exposed to live Akk11 at concentrations of 10^7^, 10^8^, and 10^9^ AFU/mL for 4 and 8 h. LDH release was measured spectrophotometrically at 490 nm, and relative activity (%) was calculated using positive and negative controls. The percentage of relative activity was calculated to the Equation (1):

(1) $$\begin{array}{c} \text{Relative activity}\;\left(\%\right)=100\\ \;\times \left(\;1-\frac{\left(\text{Experimental}\;\mathrm{LDH}\;\text{release}-\text{Medium background}\right)}{\left(\text{Maximum}\;\mathrm{LDH}\;\text{Release Control}-\text{Medium Background}\right)}\right) \end{array}$$

### 2.5 In Vivo Toxicological Evaluation

The 14‐day acute oral toxicity test, bacterial reverse mutation assay, and mammalian erythrocyte micronucleus test were performed in accordance with GB 15193.3‐2014, GB 15193.4‐2014, and GB 15193.5‐2014, respectively. All tests complied with the corresponding OECD guidelines, including OECD 423, OECD 471, and OECD 474, with appropriate modifications to align with national standards. Furthermore, a 90‐day sub‐chronic oral toxicity study was conducted completely following OECD 408 guidelines to establish the safety of prolonged Akk11 consumption. This study was performed in an OECD Good Laboratory Practice (GLP)‐certified laboratory. Unless otherwise specified, the selection of intervention doses, group size, and sex distribution in the toxicological studies was strictly conducted in accordance with the requirements of the GB and OECD guidelines. All experimental animals were required to be healthy, disease‐free, SPF‐grade rats or mice. Prior to the start of the experiment, all animals underwent health screening, including assessments of respiration, fur condition, and body weight, to ensure they met the inclusion criteria. No significant differences were observed between the treatment and control groups during the baseline period in terms of body weight, food intake, or water consumption, ensuring comparability between groups. Baseline data were recorded primarily to confirm the animals' health status before intervention and to serve as a reference for subsequent toxicity assessments.

The test material used across all studies was lyophilized Akk11 powder containing both viable and non‐viable cells, with approximately 20%–30% non‐viable cell content. This proportion was quantified live/dead proportions by flow cytometry using a dual‐stain viability kit (SYTO9/PI), with heat‐killed (pasteurized) and freshly cultured controls to set gates. This composition is typical of what is commonly found in probiotic preparations. Loss of culturability during lyophilization and early storage commonly yields non‐viable fractions on the order of approximately 10%–40%, varying by strain, protectants, and process parameters (Cabello‐Olmo et al. 2020). The 20%–30% non‐viable fraction observed in Akk11 is consistent with the range reported for commercial probiotic formulations.

#### 2.5.1 Acute Oral Toxicity Test

A 14‐day acute oral toxicity test was conducted in specific‐pathogen‐free (SPF) ICR mice (*n* = 20; 10 males and 10 females; body weight 18–22 g), obtained from Shanghai Shenchang Biotechnology Co. Ltd. The dose of 2 × 10^10^ AFU/kg body weight (bw) is based on multiple references (Gu et al. 2024; Wu et al. 2024; Dong et al. 2024), which observed no toxic effects at this dose. The animals were randomly assigned and fasted for 16 h with free access to water prior to administration. Lyophilized Akk11 powder (0.2 g) was suspended in 20 mL of sterile deionized water and administered by gavage at a limit dose of 2 × 10^10^ AFU/kg bw. The gavage volume was set at 0.2 mL/10 g body weight. Animals were observed twice daily for clinical signs of toxicity and behavioral changes. Body weights were recorded every 3 days throughout the observation period. At the end of the study, gross necropsies were performed on all animals, including any found dead during the observation period and all surviving animals after 14 days.

#### 2.5.2 Bacterial Reverse Mutation Assay

The bacterial reverse mutation assay (Ames test) was conducted using five *Salmonella typhimurium* strains: TA97a, TA98, TA100, TA102, and TA1535, in accordance with GB 15193.4‐2014 and OECD 471 guidelines. For test substances without bacterial toxicity, the recommended maximum dose in guidelines is 5 mg/plate. A geometric dose spacing factor of $\sqrt{10}$ was used to design the dose levels: 0.05 mg, 0.158 mg, 0.500 mg, 1.580 mg, and 5.000 mg per plate. The specification for Akk11 powder is 1 × 10^11^ AFU/g. Therefore, the corresponding number of live bacteria for the Akk11 experimental group at different dosages are as follows: 5 × 10^6^ AFU, 1.58 × 10^7^ AFU, 5 × 10^7^ AFU, 1.58 × 10^8^ AFU, 5 × 10^8^ AFU. The plate incorporation method was employed, with top agar supplemented with histidine and biotin. Lyophilized Akk11 powder was dissolved in sterile water and sterilized by filtration. The test sample were prepared and tested in triplicate for each strain, both with and without metabolic activation using a 10% S9 mix. For each plate, 0.1 mL of overnight bacterial culture, 0.1 mL of test sample, and 0.5 mL of S9 mix (where applicable) were added to 2 mL of molten top agar, poured onto bottom glucose agar plates, and incubated at 37°C for 48 h. After incubation, revertant colonies were counted. A result was considered positive if there was a concentration‐related increase in revertant colony numbers across the tested range and/or a reproducible increase in revertant colonies at one or more concentrations, in at least one strain, with or without metabolic activation.

#### 2.5.3 Mammalian Erythrocyte Micronucleus Test

The mammalian erythrocyte micronucleus test was conducted in accordance with GB 15193.5‐2014 and OECD 474 guidelines: Specific‐pathogen‐free (SPF) ICR mice (*n* = 10; 5 males and 5 females; body weight 25–30 g), doses were based on fractions of the LD_50_, specifically 1/2, 1/4, and 1/8. The tested doses were 2.5 × 10^9^ AFU/kg bw, 5 × 10^9^ AFU/kg bw, and 1 × 10^10^ AFU/kg bw. Animals were randomly assigned to control and test groups, receiving lyophilized Akk11 powder suspension by gavage at 0.2 mL/10 g body weight. Two administrations were performed at 24‐h intervals. Six hours after the second administration, animals were euthanized, and bone marrow was harvested from the femur. Smears were prepared, fixed with methanol, and stained with Giemsa. For each animal, 2000 immature erythrocytes were examined under a microscope, and the frequency of micronucleated immature erythrocytes was recorded. No statistically significant increase in micronucleated immature erythrocytes was observed in the Akk11‐treated group compared to the control group.

#### 2.5.4 90‐Day Sub‐Chronic Toxicity Study

A 90‐day repeated‐dose oral toxicity study was performed in accordance with OECD Guideline 408 under Good Laboratory Practice (GLP) conditions. A total of 100 specific‐pathogen‐free (SPF) Sprague–Dawley rats (Crl:CD (SD) IGS; 50 males and 50 females; 7–9 weeks old at study initiation; body weight: males 230–380 g, females 175–255 g) were obtained from Vital River Laboratory Animal Technology Co. Ltd. (SCXK (Jing) 2021‐0011). Animals were housed in an AAALAC‐accredited facility, and all experimental procedures were approved by the Institutional Animal Care and Use Committee (IACUC No.: 24‐473) of Pharmaron (Beijing) TSP Services Limited. Environmental enrichment was provided throughout the study in accordance with facility policies.

In this study, Sprague–Dawley rats were randomly assigned into four groups: control, low‐dose (1.0 × 10^11^ AFU/kg body weight/day), medium‐dose (3.0 × 10^11^ AFU/kg body weight/day), and high‐dose (9.0 × 10^11^ AFU/kg body weight/day), with 20 animals per group (10 males and 10 females). In addition, satellite groups were established for both the control and high‐dose groups, each consisting of 10 rats (5 males and 5 females). These satellite groups were included to evaluate potential changes during the recovery phase following 90 days of continuous administration.

The test material was lyophilized Akk11 powder containing approximately 20%–30% non‐viable cells, dissolved in sterilized water for injection, and administered via oral gavage at a fixed dose volume of 20 mL/kg/day. Throughout the in‐life phase, all animals were monitored for clinical signs, body weight, food consumption, ophthalmological changes, functional observational bakery (FOB) results, grip strength, and rectal temperature. Hematology, clinical chemistry (including thyroid hormones T3, T4, TSH), coagulation parameters, urinalysis, and fecal collection were performed at scheduled intervals. At study termination and after the recovery period, animals were subjected to gross necropsy, organ weight measurement, and histopathological examination. Target tissues were preserved and analyzed according to standard protocols. All evaluations were performed blinded to treatment groups. The study was conducted in compliance with both OECD and US FDA GLP requirements. All records were retained per regulatory standards.

### 2.6 Statistical Analysis

Statistical analyses were carried out using R (Version: 4.3.2). Data are presented as mean ± standard deviation, based on three independent experiments. Single‐factor analysis of variance (ANOVA) followed by Tukey's post hoc test was employed for between‐group comparisons. Group differences were assessed using one‐way ANOVA, and Tukey's post hoc test was used for multiple between‐group comparisons. Statistical significance was set at a threshold of *p* < 0.05. Significant differences are indicated as follows: *p < 0.05, **p < 0.01, ***p < 0.001.

## 3 Results

### 3.1 Isolation and Characterization of Akk11

**FIGURE 1:**
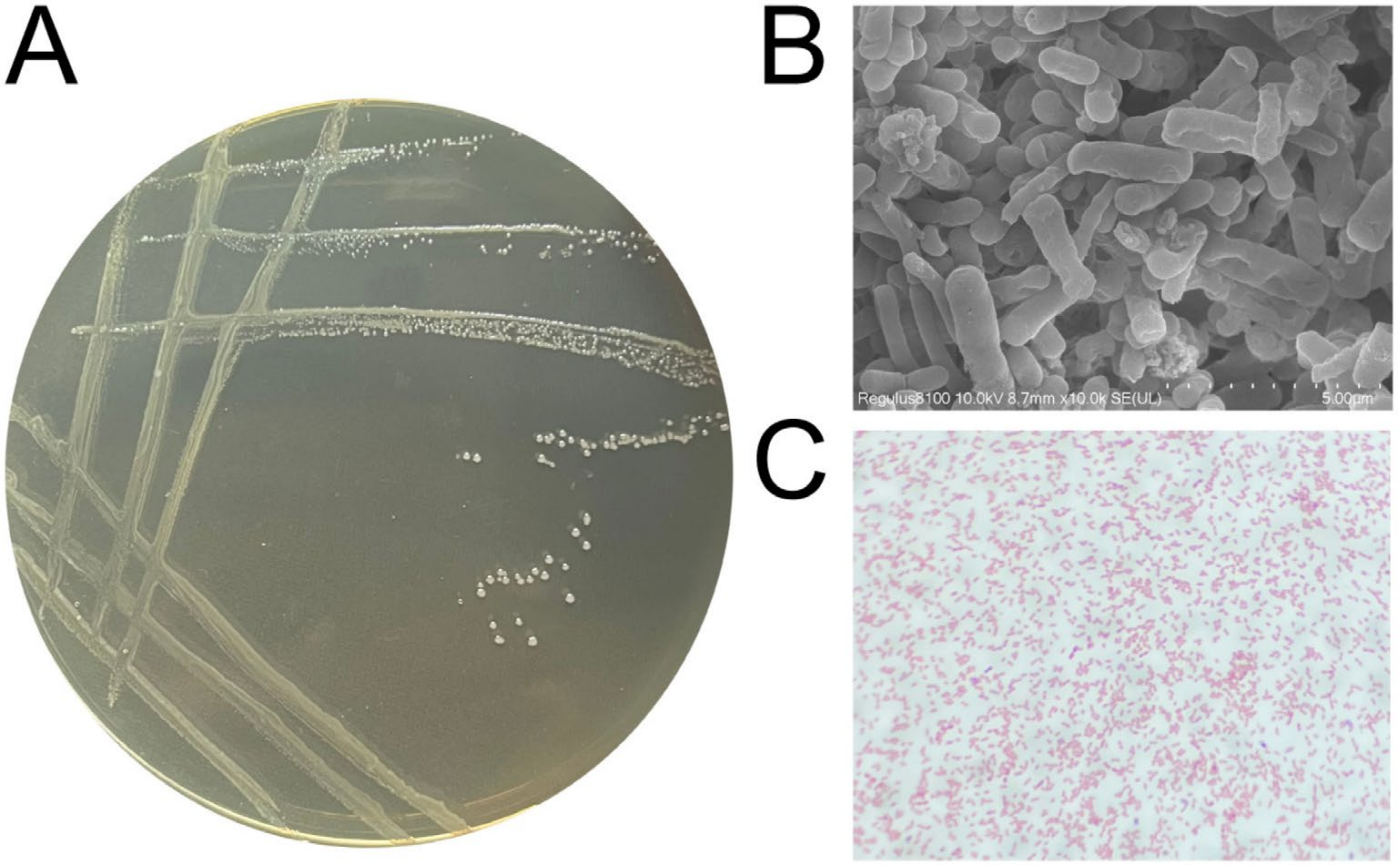
Phenotypical characterization of Akk11. (A) Bacterial colonies on agar medium. (B) Morphological characteristics of Akk11 bacterial cells under a scanning electron microscope (SEM) at 10,000× magnification. (C) Gram staining result of Akk11 at 1600× magnification.

**FIGURE 2:**
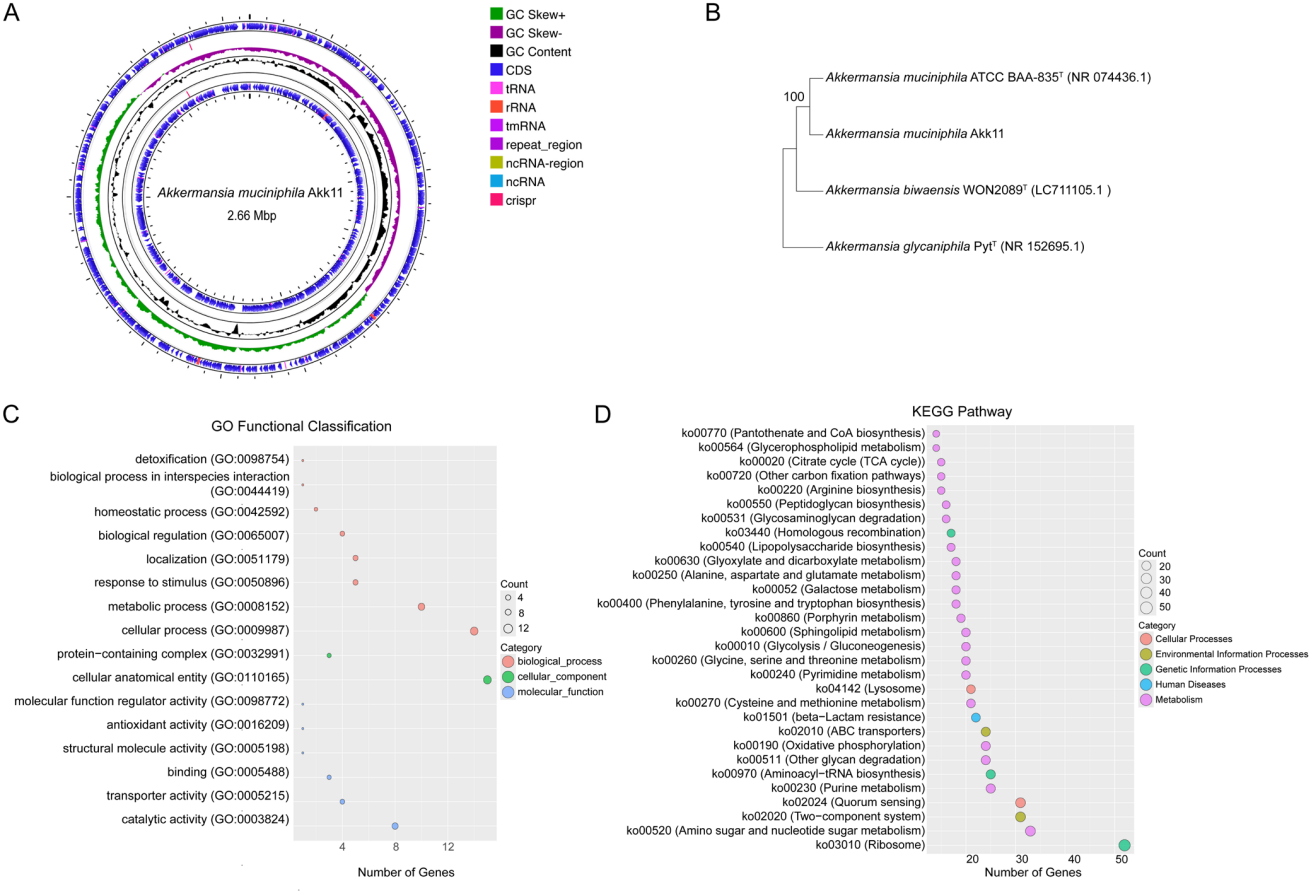
Genetic characteristics of Akk11. (A) Genome map of Akk11 completion map sequencing. (B) Phylogenetic tree of Akk11. (C) Gene ontology (GO) annotation of strain Akk11, detailing the biological processes, cellular components, and molecular functions associated with the genes. (D) Kyoto Encyclopedia of Genes and Genomes (KEGG) pathway annotation of strain Akk11, showing the integration of genomic information with functional pathways.

Strain Akk11 was originally isolated from infant feces and is currently maintained at the China Center for Type Collection (CCTCC, Deposit Number: CCTCC M 2024119), American Type Culture Collection (ATCC, Deposit Number: PTA‐127863) and Leibniz Institute DSMZ‐German Collection of Microorganisms and Cell Cultures GmbH (DSMZ, Deposit Number: DSM 35205). The strains were cultured in BHI medium added with mucin under anaerobic conditions at 37°C. After 72 h of culture, as shown in Figure 1A–C, the colonies were white, round, with a moist surface, opaque and neat edges. The bacteria were oval and short rod‐shaped with Gram‐negative characteristics, as observed using a SEM and an optical microscope. *A. muciniphila* , represented by the type strain *A. muciniphila* Muc^T^, is considered a highly abundant mucus‐degrading strain from a healthy adult (Derrien et al. 2004; Cani et al. 2022). The SDS‐PAGE electrophoresis map (Figure S1) showed that compared with the culture medium without Akk11, the mucin bands in the Akk11 culture medium were reduced, indicating Akk11 was capable of degrading mucin. Genomic analysis of Akk11 showed that the genome size of Akk11 is 2.66 Mbp with a guanine‐cytosine (GC) content of 56%, excluding plasmids, as shown in Figure 2A. ANI analysis indicated a genomic similarity of 98.36% with *A. muciniphila* Muc^T^. The phylogenetic tree showed that Akk11 and *A. muciniphila* Muc^T^ were on the same branch (Figure 2B). These results demonstrated that Akk11 is classified as *A. muciniphila* .

The GO functional and KEGG pathway annotation of Akk11 genes, presented in Figure 2C,D, respectively. Most genes are primarily involved in the Cellular Component and Biological Process categories, genetic and metabolic pathways.

### 3.2 Genotypic Assessment of AMR Genes, Virulence Factors and Toxic Production

Screening of Akk11 using the ResFinder databases did not reveal any genes associated with antibiotic resistance. The AMRFinder analyzed a potential *blaLUS‐1* gene at genomic positions 229,114–229,998 bp encoding class A beta‐lactamase, and the CARD database annotated an *adeF* gene encoding resistance‐nodulation‐cell division (RND) antibiotic efflux pump system at genomic positions 272,644–275,814 bp. This result is based on default parameters. However, according to the parameters suggested by EFSA guideline (EFSA 2024), with sequence identity > 80% and length coverage > 70%, neither of AMR genes was annotated in Akk11 or *A. muciniphila* MucT. Both genes are frequently found in Gram‐negative bacteria (Philippon et al. 2019; Filardi et al. 2022). The presence of these genes in *A. muciniphila* Muc^T^ (Turck et al. 2021; Filardi et al. 2022) is further evidence that these risk genes may be intrinsic to *A. muciniphila* . Mobile genetic elements (MGEs), including plasmids, insertion sequences (IS) and integrons analysis with ISfinder, BLASTn and VRprofile2 showed that in Akk11 there are no MGEs around AMR genes. Combined with the absence of plasmids, these results suggest a negligible risk of AMR gene transfer in Akk11.

In addition, no potentially virulence‐related genes, pathogenicity genes or candidate genes for the biosynthesis of biogenic amines were identified in Akk11 using the VirulenceFinder database, PathogenFinder screening and the HMMER software package, respectively. The VFDB database annotated the elongation factor Tu (*Ef‐Tu*) gene at genomic position 1,248,113–1,249,294 bp.

### 3.3 Phenotypic Assessment of Antibiotic Susceptibility, Biogenic Amines, D/L‐Lactic Acid Production and Hemolytic Activity of Akk11

To fully address the antibiotic resistance profile of Akk11, we performed a phenotypic test based on MICs determination for a selected group of antibiotics as shown in Table 1. According to the EFSA guidelines (EFSA Panel on Additives and Products or Substances used in Animal Feed et al. 2018), for Gram‐negative bacteria the antibiotics tested should be those for *Enterobacteriaceae*, including ampicillin, gentamicin, kanamycin, streptomycin, tetracycline, ciprofloxacin, colistin and fosfomycin. Both Akk11 and *A. muciniphila* Muc^T^ showed low sensitivity to aminoglycosides (gentamicin, kanamycin and streptomycin) and ciprofloxacin, revealing the intrinsic resistance of Akk11 to these antibiotics.

**TABLE 1:** Minimum inhibitory concentration (MICs) (mg/L) of tested antibiotics for comparison.

Strain	Ampicillin	Gentamicin	Kanamycin	Streptomycin	Tetracycline	Ciprofloxacin	Colistin	Fosfomycin
*Enterobacteriaceae* cut‐off values (EFSA Panel on Additives and Products or Substances used in Animal Feed et al. 2018)	8	2	8	16	8	0.06	2	8
*A. muciniphila* MucT (Filardi et al. 2022)	4	256	512	256	< 2	128–256	< 2	< 4
Akk11	2	128	128	128	1	64	0.25	8

Biogenic amines (tryptamine, putrescine, cadaverine, histamine, spermidine, or spermine) were not detected, with concentrations all below the method detection limit of 10 μg/L in Akk11 fermentation broth. D‐ and L‐lactic acid were also undetectable, below 150 mg/L. On Columbia blood agar plates, no hemolytic zones were observed around the growing colonies of Akk11, indicating that Akk11 is non‐hemolytic.

### 3.4 Stress Tolerance of Akk11 in Simulated Gastrointestinal Environment and Its Cytotoxicity to Mammalian Epithelial Cells

Akk11 has been studied for its ability to cope with low pH, pepsin‐containing gastric juice in the stomach, as well as trypsin and bile salts in the intestine. As shown in Table 2, the survival rate of Akk11 after 2 h incubation with modified BHI (pH 2.5), artificial gastric juice (pH 3.5), and artificial intestinal juice (pH 8) was over 99%. 96.27% of Akk11 survived for 3 h when cultured in modified BHI with 0.3% bovine bile salts. The results underline the high adaptability of Akk11 to simulated gastrointestinal environments.

**TABLE 2:** The pH 2.5, artificial gastric, intestinal and bile salt tolerance test results of *Akkermansia muciniphila* Akk11.

Stress condition	lg AFU/mL (mean ± SD, g)		Survival rate/%
	Initial	Final	
pH 2.5	8.792 ± 0.007	8.785 ± 0.021	99.92
Artificial gastric fluid (pH = 3.5)	8.803 ± 0.024	8.800 ± 0.014	99.96
Artificial intestinal fluid (pH = 8)	8.785 ± 0.007	8.763 ± 0.022	99.75
Bile salt concentration (0.3%)	8.813 ± 0.007	8.484 ± 0.007	96.27

*Note:* Akk11 was cultured in pH 2.5, artificial gastric (pH 3.5), and intestinal fluids (pH 8) for 2 h, with bile salts incubated for 3 h.

Abbreviations: AFU, active fluorescent unit; SD, standard deviation.

**FIGURE 3:**
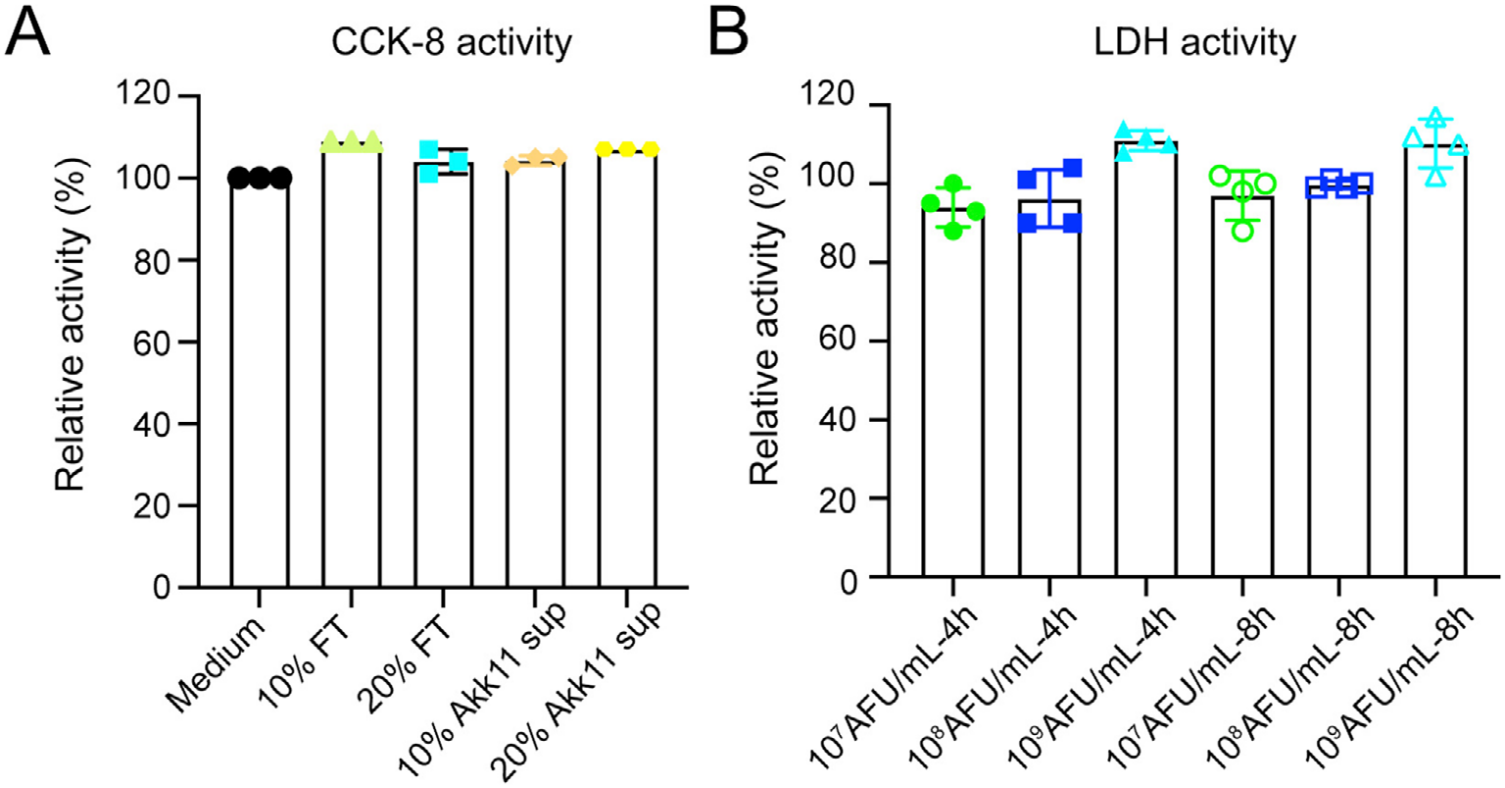
Relative activity (%) of Caco‐2 cells after incubation with Akk11 by detection of CCK‐8 activity (A) and lactate dehydrogenase (LDH) activity (B). Medium: McCoy's 5A medium control; FT: uncultured medium; sup: culture supernatant of Akk11; AFU: active fluorescent unit. The relative activity (%) for the LDH assay was calculated using Equation (1).

In addition, both the Akk11 fermentation broth and their bacteria showed no toxicity to Caco‐2 cells, as shown in Figure 3A,B.

### 3.5 Oral Toxicity and Genotoxicity of Akk11

In the in vivo acute oral toxicity test, no significant signs of toxicity or fatalities were recorded in either male or female animals during the observation period (Table 3), with typical body weight increasing without any noticeable abnormalities. A comprehensive pathological examination of all surviving animals after 14 days revealed no significant findings, which suggests that the LD_50_ of Akk11 is higher than 2 × 10^10^ AFU/kg.

**TABLE 3:** Acute oral toxicity test results of *Akkermansia muciniphila* Akk11 on mice.

Dose (AFU/kg)	Sex	Weight (mean ± SD, g)		
		Day 0	Day 7	Day 14
2 × 10^10^	Male (*n* = 10)	19.7 ± 0.67	25.7 ± 0.67	30.2 ± 0.92
	Female (*n* = 10)	20.0 ± 0.67	27.9 ± 0.99	35.9 ± 1.37

Abbreviations: AFU, active fluorescent Unit; SD, standard deviation.

The bacterial reverse mutation assay (Table 4) confirmed that both the negative and positive controls were valid. Furthermore, Akk11 did not show any mutagenic activity across the *Salmonella typhimurium* TA97a, TA98, TA100, TA102, and TA1535. According to the in vivo mammalian cell micronucleus assay results (Table 5), Akk11 did not cause any increase in the frequency of micronucleated cells. These findings suggest that Akk11 is both non‐toxic and non‐genotoxic.

**TABLE 4:** Bacterial reverse mutation assay results with and without metabolic activation of *Akkermansia muciniphila* Akk11.

Group		Dose	Number of revertant colonies (mean ± SD)				
			TA97a	TA98	TA100	TA102	TA1535
−S9	Blank control	/	172.0 ± 6.2	40.0 ± 4.6	172.3 ± 7.0	256.0 ± 6.6	17.7 ± 1.5
	Solvent control	Sterile water	172.0 ± 7.5	42.0 ± 4.6	179.7 ± 5.7	256.0 ± 9.5	16.7 ± 2.5
		DMSO	167.7 ± 8.5	42.0 ± 4.0	176.3 ± 4.5	256.0 ± 6.2	15.0 ± 3.0
	Sample	0.158 mg/plate	166.7 ± 5.0	40.3 ± 2.1	174.7 ± 6.8	246.0 ± 4.4	17.0 ± 2.0
		0.500 mg/plate	165.7 ± 10.7	43.7 ± 6.7	175.0 ± 11.4	261.3 ± 10.6	16.0 ± 2.0
		1.580 mg/plate	163.7 ± 2.1	38.3 ± 3.1	170.3 ± 7.8	261.0 ± 10.5	16.7 ± 1.5
		5.000 mg/plate	166.0 ± 9.5	37.0 ± 3.0	162.0 ± 9.6	280.7 ± 10.6	17.3 ± 1.5
	Positive control	/	1445.3 ± 75.4^a^	1063.3 ± 49.6^a^	1378.7 ± 56.6^b^	769.3 ± 17.1^a^	1147.0 ± 106.1^b^
+S9	Blank control	/	164.0 ± 5.3	37.7 ± 2.5	176.0 ± 4.6	259.7 ± 7.8	17.3 ± 2.1
	Solvent control	Sterile water	164.0 ± 6.6	43.0 ± 4.6	181.0 ± 5.6	251.0 ± 8.2	16.7 ± 2.3
		DMSO	164.3 ± 4.0	37.0 ± 2.6	172.7 ± 3.2	254.3 ± 7.0	17.7 ± 1.2
	Sample	0.158 mg/plate	171.3 ± 8.0	36.3 ± 2.5	175.0 ± 12.1	276.0 ± 5.6	17.7 ± 1.5
		0.500 mg/plate	171.7 ± 6.5	43.3 ± 4.5	180.0 ± 8.2	256.3 ± 13.4	17.0 ± 2.0
		1.580 mg/plate	166.7 ± 9.0	39.0 ± 3.0	183.3 ± 8.7	254.7 ± 6.5	16.0 ± 2.0
		5.000 mg/plate	172.7 ± 4.5	36.7 ± 5.7	176.3 ± 13.6	254.7 ± 6.5	17.7 ± 1.5
	Positive control	/	1185.7 ± 54.2^c^	1483.3 ± 79.6^c^	1274.3 ± 46.6^c^	939.7 ± 43.6^d^	1223.0 ± 107.8^e^

^a^ Dexon administered at 50.0 μg/plate.

^b^ Sodium azide administered at 1.50 μg/plate.

^c^ 2‐Aminofluorene administered at 10 μg/plate.

^d^ 1,8‐Dihydroxyanthraquinone administered at 50 μg/plate.

^e^ Cyclophosphamide administered at 20 μg/plate.

**TABLE 5:** In vivo mammalian cell micronucleus test results of *Akkermansia muciniphila* Akk11.

Group	Dose	Micronucleus rate (mean ± SD, %)	
		Female	Male
Deionized water	/	1.0 ± 0.6	1.1 ± 0.4
*Akkermansia muciniphila* Akk11	2.5 × 10^9^ AFU/kg	1.0 ± 0.4	1.2 ± 0.3
	5 × 10^9^ AFU/kg	1.3 ± 0.6	1.4 ± 0.5
	1 × 10^10^ AFU/kg	1.2 ± 0.3	1.3 ± 0.3
Cyclophosphamide (CP)	40 mg/kg	17.7 ± 2.5	19.6 ± 2.2

Abbreviations: AFU, active fluorescent unit; SD, standard deviation.

### 3.6 90‐Day Sub‐Chronic Toxicity of Akk11

The 14‐day oral toxicity study of Akk11 showed an LD_50_ greater than 2 × 10^10^ AFU/kg body weight (bw), demonstrating a high level of safety for Akk11 at high doses. Additionally, results from studies on other *Akkermansia* strains (Ma et al. 2024), with a highest dose of 5 × 10^11^ CFU/day, also support the excellent tolerance and safety of *Akkermansia* species. These findings led us to set the highest dose of Akk11 at 9 × 10^11^ AFU/kg bw/day, further confirming the safety of Akk11 at high doses in rats.

**FIGURE 4:**
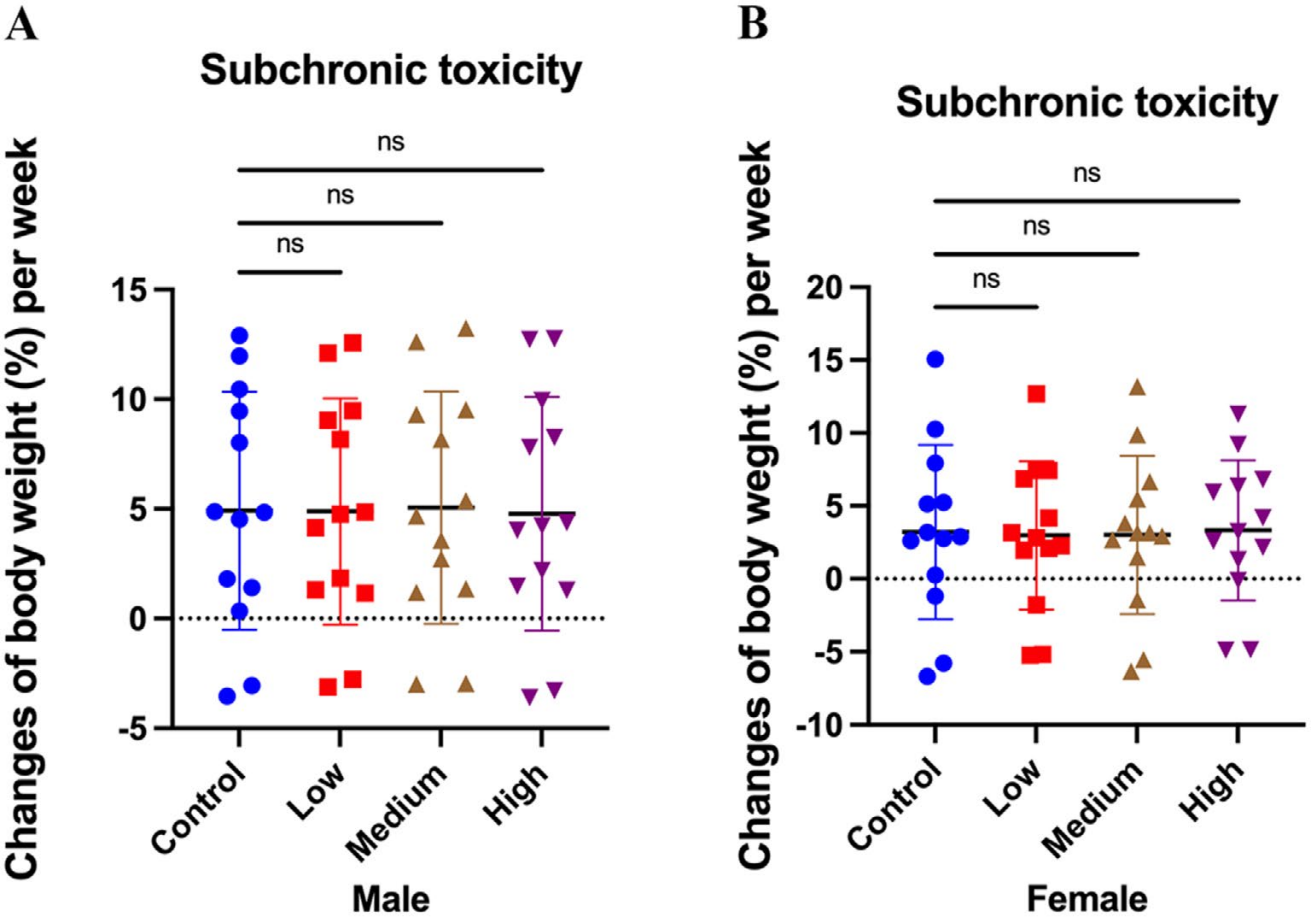
Body weight changes per week. Weekly body weight changes (%) in male (A) and female (B) rats. Control: sterile saline, low: 1.0 × 10^11^ AFU/kg body weight/day of Akk11, medium: 3.0 × 10^11^ AFU/kg body weight/day of Akk11, high: 9.0 × 10^11^AFU/kg body weight/day of Akk11.

**FIGURE 5:**
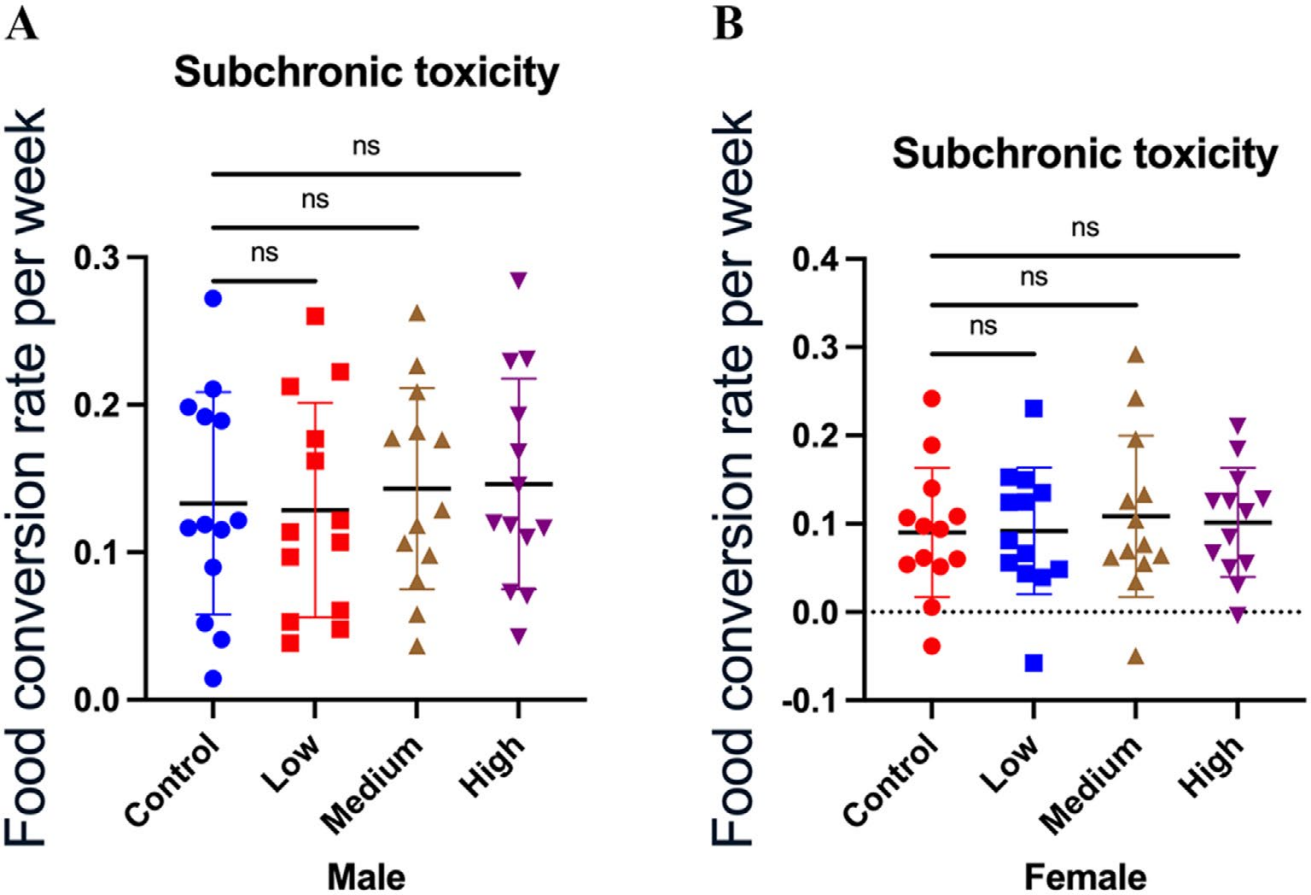
Food conversion rate per week. Weekly food conversion rate in male (A) and female (B) rats. Control: sterile saline, low: 1.0 × 10^11^ AFU/kg body weight/day of Akk11, medium: 3.0 × 10^11^ AFU/kg body weight/day of Akk11, high: 9.0 × 10^11^ AFU/kg body weight/day of Akk11.

Throughout the 90‐day sub‐chronic toxicity study, no treatment‐related adverse effects or mortality were observed in any dose group. Weekly body weight changes (Figure 4) and food conversion rates (Figure 5) showed no statistically significant differences among control and Akk11‐treated groups, indicating that Akk11 administration did not affect growth performance or nutritional intake. Among the 15 female rats in the high‐dose group, blood clotting occurred in one animal due to a procedural error during the blood sampling phase, resulting in data available from only 14 rats. However, the remaining sample size fully meets the requirements for subsequent analyses and has no impact on the experimental results.

Sporadic statistically significant differences were noted in body weight, body weight changes, food consumption, grip strength, and body temperature in the *A. muciniphila* Akk11 groups compared to the controls; however, the differences were not considered test article‐related due to their small magnitude, occurrence in a single gender only, and/or the lack of a dose–response relationship. Higher urine pH was reported on days 29, 57, and 91 in the mid‐ and high‐dose groups compared to the controls. However, this was attributed to pre‐analytical sample alterations (*i.e.,* the order of pH measurements; urine sample pH may increase over time as a result of ex vivo bacterial metabolism) and was not considered test article‐related due to the lack of correlative clinical chemistry findings. All other differences in hematology (Table 6), clinical chemistry (Table 7), coagulation, and urinalysis test results, statistically significant or not, between control and test article‐treated animals were consistent with normal variation and considered incidental due to the small magnitude, lack of dose relationship, direction of change, inconsistency between sexes, and/or the absence of correlative findings.

**TABLE 6:** Hematological parameters in 91st‐day sub‐chronic toxicity study in rats treated with *Akkermansia muciniphila* Akk11.

Blood routine indexes	Control		Low		Medium		High		Unit
	Male (*n* = 15)	Female (*n* = 15)	Male (*n* = 10)	Female (*n* = 10)	Male (*n* = 10)	Female (*n* = 10)	Male (*n* = 15)	Female (*n* = 14)	
Leukocyte count	7.780 ± 1.172	4.407 ± 2.072	8.274 ± 2.022	3.621 ± 1.024	8.669 ± 1.354	4.334 ± 1.560	7.883 ± 1.157	4.661 ± 2.182	10^9^/L
Neutrophils (Absolute)	1.565 ± 0.533	0.637 ± 0.223	1.817 ± 0.728	0.601 ± 0.177	1.753 ± 0.421	0.665 ± 0.284	1.970 ± 1.167	0.902 ± 0.808	10^9^/L
Lymphocytes (Absolute)	5.273 ± 0.756	3.297 ± 1.681	5.442 ± 1.643	2.655 ± 0.906	5.834 ± 1.062	3.230 ± 1.264	5.027 ± 1.130	3.219 ± 1.383	10^9^/L
Monocytes (Absolute)	0.797 ± 0.248	0.385 ± 0.205	0.849 ± 0.299	0.289 ± 0.098	0.903 ± 0.167	0.345 ± 0.147	0.737 ± 0.196	0.424 ± 0.263	10^9^/L
Basophils (Absolute)	0.019 ± 0.008	0.012 ± 0.009	0.023 ± 0.011	0.011 ± 0.007	0.027 ± 0.008	0.011 ± 0.007	0.023 ± 0.011	0.012 ± 0.007	10^9^/L
Eosinophils (Absolute)	0.126 ± 0.045	0.077 ± 0.038	0.143 ± 0.030	0.065 ± 0.022	0.152 ± 0.058	0.083 ± 0.046	0.127 ± 0.034	0.104 ± 0.052	10^9^/L
Platelet count	1040.5 ± 121.6	926.1 ± 95.4	1074.0 ± 79.7	986.0 ± 76.6	1074.0 ± 106.9	955.2 ± 95.8	1062.5 ± 137.4	1020.4 ± 114.1	10^9^/L
Mean platelet volume	7.12 ± 0.32	7.01 ± 0.23	7.11 ± 0.17	7.01 ± 0.20	6.92 ± 0.12	6.97 ± 0.20	6.97 ± 0.16	7.00 ± 0.18	fL
Erythrocyte count	9.239 ± 0.528	8.005 ± 0.649	8.915 ± 0.148	7.820 ± 0.318	9.268 ± 0.376	7.942 ± 0.492	9.166 ± 0.412	8.136 ± 0.416	10^12^/L
Hematocrit	46.89 ± 2.33	43.95 ± 3.67	45.02 ± 1.38	42.49 ± 1.88	46.84 ± 1.68	43.34 ± 2.66	47.25 ± 2.45	44.35 ± 2.92	%
Hemoglobin	16.12 ± 0.74	15.07 ± 1.12	15.48 ± 0.52	14.63 ± 0.66	16.13 ± 0.56	14.95 ± 0.96	16.15 ± 0.78	15.06 ± 0.84	g/dL
Mean corpuscular volume	50.80 ± 1.29	54.89 ± 1.22	50.49 ± 1.12	54.36 ± 1.59	50.55 ± 1.02	54.60 ± 1.59	51.55 ± 0.87	54.50 ± 1.61	fL
Mean corpuscular hemoglobin	17.47 ± 0.60	18.83 ± 0.47	17.36 ± 0.45	18.70 ± 0.53	17.41 ± 0.40	18.83 ± 0.61	17.61 ± 0.25	18.51 ± 0.52	pg
Mean corpuscular hemoglobin concentration reticulocyte	34.38 ± 0.41	34.32 ± 0.44	34.49 ± 0.38	34.43 ± 0.24	34.44 ± 0.31	34.50 ± 0.37	34.18 ± 0.47	33.97 ± 0.64	g/dL
Reticulocyte (Absolute)	233.14 ± 27.47	224.11 ± 40.10	236.51 ± 18.41	200.24 ± 25.86	234.31 ± 24.20	194.40 ± 18.64	235.07 ± 45.87	219.21 ± 53.57	10^9^/L

*Note:* Data are presented as mean ± standard deviation. The low, medium and high dose groups are treated with 1.0 × 10^11^, 3.0 × 10^11^, or 9.0 × 10^11^ AFU/kg body weight/day of Akk11, respectively.

**TABLE 7:** Biochemical indicators in 91st‐day sub‐chronic toxicity study in rats.

Biochemical indicators	Control		Low		Medium		High		Unit
	Male (*n* = 15)	Female (*n* = 15)	Male (*n* = 10)	Female (*n* = 10)	Male (*n* = 10)	Female (*n* = 10)	Male (*n* = 15)	Female (*n* = 14)	
Alanine aminotransferase	32.1 ± 4.9	33.5 ± 19.0	30.6 ± 8.4	29.3 ± 11.9	28.9 ± 3.1	25.1 ± 5.5	28.2 ± 4.8	30.4 ± 11.8	U/L
Aspartate aminotransferase	136.5 ± 26.3	138.1 ± 28.1	137.8 ± 35.1	110.5 ± 19.8	132.8 ± 9.1	123.7 ± 30.0	130.2 ± 25.5	112.5 ± 34.7*	U/L
Alkaline phosphatase	73.2 ± 16.3	33.2 ± 8.0	76.5 ± 16.1	32.3 ± 4.8	80.7 ± 13.8	31.1 ± 5.6	77.1 ± 15.0	34.3 ± 7.9	U/L
Creatine phosphate kinase	640.0 ± 272.5	516.5 ± 199.7	617.9 ± 258.0	381.1 ± 157.3	615.4 ± 115.9	478.1 ± 198.2	569.1 ± 177.8	426.1 ± 204.1	U/L
lactate dehydrogenase	2459.44 ± 1146.12	1646.39 ± 638.30	2578.49 ± 1108.07	1227.98 ± 620.46	2478.72 ± 448.18	1893.42 ± 1121.89	2314.59 ± 910.71	1261.26 ± 705.97	U/L
Total protein	62.57 ± 4.66	67.77 ± 6.97	59.31 ± 1.18	67.69 ± 4.11	59.78 ± 1.56	68.29 ± 4.51	62.87 ± 5.12	70.69 ± 7.01	g/L
Albumin	42.40 ± 2.65	50.99 ± 5.51	40.40 ± 1.15	51.35 ± 3.70	41.32 ± 1.32	51.78 ± 4.43	42.89 ± 2.96	53.25 ± 6.06	g/L
Globulin	20.17 ± 2.47	16.77 ± 2.46	18.91 ± 1.07	16.34 ± 1.32	18.46 ± 1.55	16.51 ± 2.12	19.98 ± 2.94	17.45 ± 1.98	g/L
Albumin/Globulin ratio	2.123 ± 0.211	3.081 ± 0.415	2.144 ± 0.161	3.158 ± 0.323	2.257 ± 0.239	3.194 ± 0.560	2.180 ± 0.297	3.077 ± 0.384	Ratio
Blood urea	5.475 ± 0.776	6.291 ± 1.027	5.662 ± 0.631	6.104 ± 0.987	5.353 ± 0.612	6.229 ± 0.895	5.617 ± 0.923	6.371 ± 1.263	mmol/L
Creatinine	32.6 ± 3.4	40.8 ± 4.3	31.0 ± 1.4	38.5 ± 4.1	30.7 ± 3.9	37.8 ± 4.8	30.9 ± 3.8	41.6 ± 6.4	μmol/L
BUN/Creatine ratio	41.895 ± 6.024	38.229 ± 4.323	45.342 ± 5.128	39.354 ± 5.109	43.623 ± 5.469	40.965 ± 4.409	45.353 ± 7.388	38.107 ± 5.328	Ratio
Glucose	5.836 ± 0.505	5.957 ± 0.916	5.979 ± 0.359	5.979 ± 0.517	6.164 ± 0.537	6.404 ± 0.814	5.840 ± 0.579	6.452 ± 1.445	mmol/L
Cholesterol	1.734 ± 0.307	2.062 ± 0.470	1.605 ± 0.421	1.911 ± 0.371	1.528 ± 0.211	2.090 ± 0.428	1.554 ± 0.308	1.904 ± 0.415	mmol/L
Triglycerides	0.403 ± 0.228	0.248 ± 0.143	0.420 ± 0.239	0.220 ± 0.139	0.474 ± 0.140	0.189 ± 0.044	0.409 ± 0.197	0.239 ± 0.173	mmol/L
Sodium	145.39 ± 1.81	143.43 ± 1.58	144.22 ± 0.48	142.13 ± 0.68*	144.77 ± 0.64	142.35 ± 0.72	145.35 ± 1.47	142.77 ± 1.06	mmol/L
Potassium	4.906 ± 0.607	4.267 ± 0.440	4.612 ± 0.335	4.144 ± 0.211	4.609 ± 0.149	4.025 ± 0.328	4.283 ± 0.631	4.389 ± 0.556	mmol/L
Chloride	104.53 ± 1.20	104.05 ± 2.09	104.92 ± 1.40	104.33 ± 1.70	105.31 ± 1.15	104.15 ± 1.54	103.90 ± 1.64	102.84 ± 1.44	mmol/L
Calcium	2.473 ± 0.118	2.511 ± 0.149	2.362 ± 0.037**	2.451 ± 0.090	2.423 ± 0.047	2.465 ± 0.075	2.461 ± 0.121	2.551 ± 0.136	mmol/L
Phosphorus	2.07 ± 0.21	1.74 ± 0.37	1.88 ± 0.21	1.55 ± 0.32	1.95 ± 0.20	1.60 ± 0.33	2.07 ± 0.21	1.71 ± 0.39	mmol/L
Hight density lipoprotein cholesterol	0.903 ± 0.165	1.159 ± 0.253	0.835 ± 0.177	1.079 ± 0.204	0.773 ± 0.119	1.171 ± 0.243	0.824 ± 0.155	1.109 ± 0.212	mmol/L
Low density lipoprotein cholesterol	0.484 ± 0.138	0.374 ± 0.136	0.431 ± 0.183	0.321 ± 0.080	0.380 ± 0.078	0.361 ± 0.129	0.404 ± 0.127	0.301 ± 0.115	mmol/L

*Note:* Data are presented as mean ± standard deviation. The low, medium and high dose groups are treated with 1.0 × 10^11^, 3.0 × 10^11^, or 9.0 × 10^11^ AFU/kg body weight/day of Akk11, respectively. *p < 0.05, compared with the data of the control group of the corresponding sex.

Statistically significant increases in mean absolute spleen weight and spleen to body weight ratios were reported in males of the low‐, mid‐, and high‐dose groups compared to the controls (Table 9). Similarly, a statistically significant increase in spleen to brain weight ratio was reported for males of the low‐ and mid‐dose groups compared to the controls. However, the effect did not display any dose–response relationship (*i.e.,* the magnitude of the effect had an inverse relationship with *A. muciniphila* Akk11 consumption such that the most prominent effect was reported in the low‐dose group), and this effect did not persist during the recovery interval. In addition, no significant differences were reported in females. As such, these effects are attributed to biological variability and not considered to be a test article‐related adverse effect. Increased macrophage cellularity in the red pulp of the spleen, characterized by a higher number of large macrophages, with abundant cytoplasm, and blunted spleen edges, was also reported in 2/10 and 4/10 males of the low‐ and high‐dose groups, respectively. However, no bacteria were identified upon H&E evaluation, no correlative findings were reported in hematological parameters, no findings of extramedullary hematopoiesis (a clear sign of immune‐mediated myeloproliferation) were reported, and no microscopic findings were reported in females. Furthermore, no increase in macrophage cellularity was reported after the recovery interval, demonstrating that any effect was clearly reversible. Taken together, this finding was considered an adaptive immune response, similar to those described by den Haan and Kraal (2012) and Borges da Silva et al. (2015), rather than a test article‐related adverse effect. No other significant and/or test article‐related effects were reported in any measured parameter during the 90‐day study period.

After the 90‐day gavage period, an additional 28‐day observation of the control and high‐dose groups revealed no adverse conditions in the rats during the recovery phase. Moreover, no significant differences were observed between the control and high‐dose groups in hematological and biochemical parameters (Tables 10, 11, 12), and minor variations were considered unrelated to dose dependency. Histopathological examination of major organs revealed no abnormalities or treatment‐related lesions. Collectively, these findings demonstrate that oral administration of Akk11, in the form of lyophilized powder containing both viable and non‐viable cells (with approximately 20%–30% non‐viable cells), at doses up to 9 × 10^11^ AFU/kg/day for 90 consecutive days, is well‐tolerated and does not induce toxicological effects under the tested conditions.

## 4 Discussion

Public interest in probiotic functionality continues to rise, and *Akkermansia muciniphila* has emerged as a next‐generation candidate with diverse host‐modulatory potential (Cani et al. 2022). Across observational and experimental literature, higher *A. muciniphila* abundance generally associates inversely with metabolic dysfunction, consistent with putative anti‐inflammatory and barrier‐supporting effects (Liu et al. 2021). These converging signals motivate strain‐level evaluation while avoiding overinterpretation of causality.

Pasteurized *A. muciniphila* , has been authorized in the EU as a novel food (Turck et al. 2021), and preclinical studies together with a proof‐of‐concept human trial provide initial evidence supporting a favorable safety profile in defined contexts (Cani et al. 2022). At the same time, *A. muciniphila* is not currently on the EFSA QPS list, reflecting limited strain‐resolved evidence and remaining knowledge gaps, including questions raised in some reports related to neurological conditions (Hou et al. 2023; Ma et al. 2024; Koutsoumanis, Allende, Alvarez‐Ordonez, et al. 2020; Druart et al. 2021). Against this backdrop, we position Akk11 within established assessment frameworks (EFSA, FDA GRAS, USP, and FAO/WHO) to contribute strain‐specific data that address identity, safety, and functional plausibility (Araya et al. 2002; Boyte et al. 2024; Turck et al. 2021).

Accurate microbial identification is the first step in evaluating probiotics. Akk11 was identified as *A. muciniphila* , a Gram‐negative, mucin‐degrading commensal, showing 98.36% similarity to the type strain, consistent with species‐level assignment (Figure 1). As a mucin‐degrading specialist in the human gut, *A. muciniphila* occupies a key ecological niche but has also been discussed as a potential risk factor for host disruption in certain settings. Increased *A. muciniphila* abundance has been reported in neurological disorders, raising concerns about barrier integrity and inflammation (Radisavljevic et al. 2018). At the same time, preclinical studies suggest possible neuroprotective effects through gut–brain axis mechanisms in Parkinson's disease models (Wang et al. 2025). These mixed signals underscore the need for strain‐specific, mechanism‐linked evaluation.

Genotypic and phenotypic analyses revealed that Akk11 has a good safety profile (Figures 1, 2, 3, Tables 1 and 2). Specifically, genetic analysis annotated the presence of AMR genes responsible for encoding beta‐lactamase, an intrinsic resistance factor for aminoglycosides commonly observed in some Gram‐negative bacilli (Philippon et al. 2019; Filardi et al. 2022). The resistance genes detected in Akk11 were also identified in *A. muciniphila* Muc^T^, and there were five *A. muciniphila* strains (Sap1, Amap1, Vtp7, Rcp22, Amup9) that presented high resistance levels to aminoglycosides (Turck et al. 2021; Philippon et al. 2019), indicating that resistance is an intrinsic trait among *A. muciniphila* . Further phenotypic testing confirmed weak resistance to certain antibiotics, such as aminoglycosides (gentamicin, kanamycin and streptomycin) and ciprofloxacin. Importantly, these resistance traits are intrinsic, meaning they are naturally occurring within Akk11 and do not pose a significant safety threat. No plasmids or mobile genetic elements (MGEs) were detected around the resistance genes, suggesting that there is no risk of horizontal gene transfer, which could otherwise accelerate the spread of antibiotic resistance (De Melo Pereira et al. 2018). The annotated AMR genes is based on default parameters. However, according to the parameters suggested by EFSA guideling (EFSA. 2024) with sequence identity > 80% and length coverage > 70%, neither of AMR genes was analyzed in Akk11 or *A. muciniphila* MucT. *Ef‐Tu*, a protein abundant in bacteria and essential for translational accuracy (Widjaja et al. 2017), was detected in six other strains of *A. muciniphila* by the VFDB database. This putative virulence factor had not been reported to play a role in the pathogenicity of *A. muciniphila* (Hou et al. 2023), indicating that Akk11 is non‐toxigenic. In addition, Akk11 did not harbor virulence‐related genes associated with biogenic amines or D, L‐lactic acid production. The lack of toxic effects was further confirmed in co‐culture experiments with intestinal epithelial cells, and toxicity studies including a 14‐day acute toxicity study (Table 3), a bacterial reverse mutation assay (Table 4), an in vivo mammalian cell micronucleus test (Table 5), a 90‐day sub‐chronic toxicity study (Tables 6, 7, 8, 9, 10, 11, 12) showed no adverse effects of Akk11 on mice and rats. No dose‐dependent effects were observed in key detection indicators (body weight, food consumption, routine blood parameters, histopathology, and biochemical markers) in either male or female rats following gastric administration of Akk11 (Figures 3 and 4), indicating that the treatment does not produce dose‐related or sex‐specific impacts. Heat‐treated/non‐viable preparations have documented bioactivity in beneficial effects, mainly immunomodulatory effects, protection against enteropathogens, and maintenance of intestinal barrier integrity (Pique et al. 2019). These findings reinforce Akk11's safety and tolerance for human use, as a live or pasteurized (non‐viable) preparation.

**TABLE 8:** Organ weights in 91st‐day sub‐chronic toxicity study in rats.

Organ	Control		Low		Medium		High		Unit
	Male (*n* = 10)	Female (*n* = 10)	Male (*n* = 10)	Female (*n* = 10)	Male (*n* = 10)	Female (*n* = 10)	Male (*n* = 10)	Female (*n* = 10)	
Adrenals	0.0539 ± 0.0064	0.0706 ± 0.0123	0.0565 ± 0.0087	0.0715 ± 0.0127	0.0571 ± 0.0072	0.0708 ± 0.0093	0.0575 ± 0.0115	0.0707 ± 0.0064	g
Brain	2.2293 ± 0.1000	2.0341 ± 0.0724	2.2646 ± 0.0987	2.0623 ± 0.0797	2.2489 ± 0.0927	2.0916 ± 0.0698	2.2409 ± 0.0725	2.0415 ± 0.0957	g
Heart	1.7438 ± 0.1589	1.0822 ± 0.0658	1.8213 ± 0.1580	1.1203 ± 0.1429	1.8399 ± 0.2570	1.0700 ± 0.0560	1.8191 ± 0.2701	1.1367 ± 0.0745	g
Kidneys	3.1317 ± 0.2824	1.8633 ± 0.1928	3.2788 ± 0.2932	1.8104 ± 0.2006	3.2138 ± 0.2698	1.7920 ± 0.0811	3.2712 ± 0.3738	1.8898 ± 0.1468	g
Liver	12.1215 ± 1.3442	7.1467 ± 0.8205	12.9422 ± 1.1729	7.2375 ± 0.7589	13.2335 ± 1.9581	7.0844 ± 0.7070	12.8527 ± 2.0648	7.4114 ± 0.8382	g
Spleen	0.7174 ± 0.0314	0.5460 ± 0.0658	0.8735 ± 0.1043**	0.5679 ± 0.1089	0.8699 ± 0.1376**	0.5448 ± 0.0469	0.8483 ± 0.1538**	0.5435 ± 0.0621	g
Thymus	0.3160 ± 0.0699	0.2598 ± 0.0582	0.3715 ± 0.0533	0.3335 ± 0.0959	0.3411 ± 0.0756	0.2926 ± 0.0597	0.3189 ± 0.0791	0.3002 ± 0.0619	g
Thyroids	0.0560 ± 0.0171	0.0297 ± 0.0047	0.0595 ± 0.0262	0.0313 ± 0.0058	0.0662 ± 0.0381	0.0299 ± 0.0051	0.0468 ± 0.0113	0.0306 ± 0.0030	g
Testes	3.4362 ± 0.2590	0.7281 ± 0.1979	3.4473 ± 0.3461	0.6857 ± 0.1506	3.6407 ± 0.2889	0.6539 ± 0.1522	3.5442 ± 0.3188	0.6547 ± 0.1403	g
Epididymides	1.4610 ± 0.1418	0.0905 ± 0.0175	1.5607 ± 0.1635	0.0939 ± 0.0224	1.5960 ± 0.1867	0.0938 ± 0.0170	1.6238 ± 0.1494	0.0884 ± 0.0107	g
Lung	1.8246 ± 0.1402	1.3078 ± 0.1393	1.8311 ± 0.1511	1.3659 ± 0.1042	1.8358 ± 0.1417	1.3202 ± 0.0632	1.7227 ± 0.1535	1.3474 ± 0.0800	g

*Note:* Data are presented as mean ± standard deviation. The low, medium and high dose groups are treated with 1.0 × 10^11^, 3.0 × 10^11^, or 9.0 × 10^11^ AFU/kg body weight/day of Akk11, respectively. **p < 0.01, compared with the data of the control group of the corresponding sex.

**TABLE 9:** Spleen‐brain weight ratio and spleen‐body weight ratio in 91st‐day sub‐chronic toxicity study.

Sex	Males				Females			
Dose level (AFU/kg/day)	0	1.0 × 10^11^	3.0 × 10^11^	9.0 × 10^11^	0	1.0 × 10^11^	3.0 × 10^11^	9.0 × 10^11^
*Spleen*								
Absolute weight (g)	0.7174	0.8735**	0.8699**	0.8483**	0.5460	0.5679	0.5448	0.5435
% Body weight (ratio)	0.1365	0.1630**	0.1559*	0.1583*	0.1874	0.1928	0.1918	0.1795
% Brain weight (ratio)	32.264	38.595**	38.650*	37.809	26.803	27.510	26.096	26.727

*Note:* Values shown are group means; bolded values indicate statistical significance compared to control group means (*p < 0.05; **p < 0.01).

**TABLE 10:** Hematological parameters in 119th‐day sub‐chronic toxicity study in rats treated with *Akkermansia muciniphila* Akk11.

Blood routine indexes	Control		High		Unit
	Male (*n* = 5)	Female (*n* = 5)	Male (*n* = 5)	Female (*n* = 5)	
Leukocyte count	7.218 ± 2.014	5.075 ± 1.255	6.650 ± 1.537	5.624 ± 1.314	10^9^/L
Neutrophils (Absolute)	1.638 ± 0.367	0.720 ± 0.202	1.436 ± 0.527	1.036 ± 0.919	10^9^/L
Lymphocytes (Absolute)	4.658 ± 1.467	3.748 ± 0.958	4.35 ± 0.942	3.916 ± 0.968	10^9^/L
Monocytes (Absolute)	0.792 ± 0.326	0.505 ± 0.101	0.742 ± 0.283	0.534 ± 0.230	10^9^/L
Basophils (Absolute)	0.016 ± 0.005	0.013 ± 0.005	0.012 ± 0.004	0.012 ± 0.008	10^9^/L
Eosinophils (Absolute)	0.114 ± 0.039	0.090 ± 0.018	0.108 ± 0.013	0.126 ± 0.023*	10^9^/L
Platelet count	951.2 ± 76.8	969.5 ± 35.2	1090.4 ± 105.5*	1034.2 ± 176.7	10^9^/L
Mean platelet volume	7.14 ± 0.18	7.20 ± 0.14	7.12 ± 0.18	7.14 ± 0.30	fL
Erythrocyte count	8.966 ± 0.148	8.103 ± 0.276	8.888 ± 0.254	7.954 ± 0.506	10^12^/L
Hematocrit	44.84 ± 0.47	43.55 ± 1.11	45.68 ± 1.22	42.78 ± 2.42	%
Hemoglobin	15.26 ± 0.27	15.38 ± 0.35	15.62 ± 0.50	15.00 ± 0.95	g/dL
Mean corpuscular volume	50.04 ± 1.13	53.78 ± 1.76	51.40 ± 0.79	53.84 ± 1.77	fL
Mean corpuscular hemoglobin	17.06 ± 0.53	18.98 ± 0.68	17.56 ± 0.40	18.86 ± 0.68	pg
Mean corpuscular hemoglobin concentration reticulocyte	34.04 ± 0.38	35.30 ± 0.14	34.18 ± 0.29	35.06 ± 0.34	g/dL
Reticulocyte (Absolute)	230.42 ± 23.75	229.30 ± 29.51	235.60 ± 37.92	246.84 ± 89.90	10^9^/L

*Note:* Data are presented as mean ± standard deviation. The high dose groups are treated with 9.0 × 10^11^ AFU/kg body weight/day of Akk11, respectively. *p < 0.05, compared with the data of the control group of the corresponding sex.

**TABLE 11:** Biochemical indicators in 119th‐day sub‐chronic toxicity study in rats.

Biochemical indicators	Control		High		Unit
	Male (*n* = 5)	Female (*n* = 5)	Male (*n* = 5)	Female (*n* = 5)	
Alanine aminotransferase	38.4 ± 12.9	25.4 ± 5.0	32.0 ± 3.9	39.6 ± 17.0	U/L
Aspartate aminotransferase	170.4 ± 46.1	127.4 ± 35.3	130.0 ± 28.3	148.4 ± 22.5	U/L
Alkaline phosphatase	62.8 ± 8.7	30.4 ± 8.6	63.6 ± 11.6	23.4 ± 6.5	U/L
Creatine phosphate kinase	810.6 ± 238.0	578.6 ± 239.7	615.0 ± 253.1	491.0 ± 307.5	U/L
lactate dehydrogenase	3335.14 ± 952.80	2499.72 ± 1246.10	2446.66 ± 1161.09	1907.30 ± 1387.39	U/L
Total protein	59.22 ± 1.14	65.48 ± 2.43	59.44 ± 3.34	71.82 ± 5.60*	g/L
Albumin	40.06 ± 0.41	49.20 ± 2.46	40.76 ± 1.17	54.98 ± 5.19	g/L
Globulin	19.16 ± 1.16	16.28 ± 1.95	18.68 ± 2.73	16.84 ± 1.70	g/L
Albumin/Globulin ratio	2.100 ± 0.139	3.062 ± 0.445	2.220 ± 0.328	3.294 ± 0.475	Ratio
Blood urea	6.104 ± 0.890	5.896 ± 0.479	5.342 ± 0.746	6.724 ± 1.273	mmol/L
Creatinine	32.8 ± 5.2	37.8 ± 2.8	32.0 ± 3.7	37.2 ± 5.4	μmol/L
BUN/Creatine ratio	46.406 ± 5.254	38.988 ± 5.706	41.380 ± 2.457	44.820 ± 4.851	Ratio
Glucose	7.234 ± 0.602	6.646 ± 0.959	6.988 ± 0.882	6.662 ± 0.575	mmol/L
Cholesterol	1.530 ± 0.345	1.916 ± 0.145	1.548 ± 0.388	2.178 ± 0.560	mmol/L
Triglycerides	0.540 ± 0.164	0.314 ± 0.174	0.570 ± 0.348	0.446 ± 0.199	mmol/L
Sodium	143.42 ± 0.83	143.66 ± 1.37	144.62 ± 1.08	142.20 ± 1.24	mmol/L
Potassium	4.684 ± 0.278	4.382 ± 0.445	4.626 ± 0.340	4.340 ± 0.393	mmol/L
Chloride	105.40 ± 0.64	106.66 ± 1.36	106.38 ± 1.21	104.94 ± 1.84	mmol/L
Calcium	2.304 ± 0.018	2.416 ± 0.015	2.288 ± 0.098	2.540 ± 0.097**	mmol/L
Phosphorus	1.84 ± 0.11	1.50 ± 0.14	1.86 ± 0.09	1.44 ± 0.24	mmol/L
Hight density lipoprotein cholesterol	0.812 ± 0.206	1.126 ± 0.056	0.826 ± 0.189	1.284 ± 0.293	mmol/L
Low density lipoprotein cholesterol	0.410 ± 0.053	0.332 ± 0.060	0.438 ± 0.148	0.390 ± 0.164	mmol/L

*Note:* Data are presented as mean ± standard deviation. The high‐dose group was treated with 9.0 × 10^11^ AFU/kg body weight/day of Akk11. * *p* < 0.05, ** *p* < 0.1, compared with the data of the control group of the corresponding sex.

**TABLE 12:** Organ weights in 119th‐day sub‐chronic toxicity study in rats.

Organ	Control		High		Unit
	Male (*n* = 5)	Female (*n* = 5)	Male (*n* = 5)	Female (*n* = 5)	
Adrenals	0.0630 ± 0.0094	0.0718 ± 0.0113	0.0546 ± 0.0087	0.0650 ± 0.0017	g
Brain	2.3320 ± 0.0873	2.1042 ± 0.0943	2.1580 ± 0.1552	2.0678 ± 0.0791	g
Heart	1.8286 ± 0.2399	1.1438 ± 0.0933	1.8196 ± 0.1160	1.1484 ± 0.1862	g
Kidneys	3.3934 ± 0.2425	1.9386 ± 0.1721	3.1816 ± 0.3924	1.9106 ± 0.1435	g
Liver	13.8756 ± 2.0072	7.6920 ± 0.6251	13.4140 ± 1.7404	7.9360 ± 1.0783	g
Spleen	0.8240 ± 0.0678	0.5688 ± 0.0856	0.8186 ± 0.1132	0.5926 ± 0.0639	g
Thymus	0.2666 ± 0.0913	0.2842 ± 0.0612	0.2929 ± 0.0565	0.3180 ± 0.0662	g
Thyroids	0.0510 ± 0.0098	0.0270 ± 0.0059	0.0592 ± 0.0342	0.0274 ± 0.0049	g
Testes	3.6506 ± 0.3813	0.7958 ± 0.1996	3.5936 ± 0.2515	0.7810 ± 0.1917	g
Epididymides	1.6732 ± 0.1681	0.0886 ± 0.0049	1.6498 ± 0.1761	0.0882 ± 0.0144	g
Lung	1.8956 ± 0.0784	1.4186 ± 0.115	1.8300 ± 0.0832	1.4066 ± 0.1315	g

*Note:* Data are presented as mean ± standard deviation. The high‐dose group was treated with 9.0 × 10^11^ AFU/kg body weight/day of Akk11.

For probiotics to function effectively, they must endure various stresses imposed by the human body, such as low pH, gastric acid, and pepsin in the stomach after ingestion, as well as bile salts in the intestine. Akk11 demonstrated the ability to tolerate these stress conditions in simulated gastrointestinal environments and colonize gastro‐intestinal tract (GIT) epithelial cells (Table 2). Over 99% of Akk11 survived in simulated gastric and intestinal conditions, demonstrating its exceptional resilience compared to conventional probiotics like lactic acid bacteria (Gu et al. 2024; Chen et al. 2024). This ability to withstand gastric and bile salt conditions, coupled with its colonization of gut epithelial cells, positions Akk11 as an ideal candidate for oral probiotic supplementation.

This study provides some preliminary data on the safety of Akk11, but several limitations remain, which should be addressed and improved in future research. Firstly, the influence of confounding factors has not been fully explored. The human gut microbiota co‐evolves with the host and is influenced by various factors such as host genetics, mode of delivery, diet, environment, and medications (Ioannou et al. 2024). In particular, the growth and abundance of *A. muciniphila* are largely regulated by dietary habits, with the intake of dietary fiber and prebiotics (e.g., inulin‐type fructo‐oligosaccharides, green tea polyphenols, oat bran) significantly affecting *A. muciniphila* abundance (Everard et al. 2014; Anhê et al. 2015; Axling et al. 2012; Yang et al. 2016). Periodic fasting has been found to promote the proliferation of *A. muciniphila* by reshaping the gut microbiome (Ozkul et al. 2019). These dietary patterns or food components may directly influence the metabolism of *A. muciniphila* or indirectly regulate its growth by altering the gut microenvironment (Van Buiten et al. 2024). Moreover, the host's genetic background may influence *A. muciniphila* colonization in the gut (Johansson et al. 2008). The current study did not fully consider the potential impact of these confounding factors on Akk11's behavior in vivo. Therefore, future research should give more attention to these factors and assess their interactions in the process of Akk11's action. Secondly, the long‐term impact on gut barrier function has not been evaluated. The current study did not assess its specific effects on gut barrier integrity and long‐term impact. Some studies suggest that *A. muciniphila* improves gut barrier function by promoting goblet cell mucin secretion, thickening the mucus layer, and enhancing tight junction protein expression (e.g., through mechanisms such as AmEVs ( *A. muciniphila* ‐derived extracellular vesicles) regulating intestinal tight junction structure and reducing gut permeability) (Shin et al. 2014; Bian et al. 2019; Kang et al. 2013; Cario et al. 2007; Kim et al. 2021). However, whether these effects can be sustained over time, or whether mucin degradation could lead to increased gut permeability, is still lacking sufficient data. To better understand the long‐term impact of Akk11 on gut barrier function, future studies should include long‐term follow‐up observations and combine quantitative measurements of tight junction proteins to assess the sustained effects of Akk11 on the gut barrier. Thus, future research should further explore the physiological mechanisms of Akk11 using animal models, investigate its role in the gut microbiota and host immune system, and explore its potential as a probiotic. Clinical trials should be conducted to validate its safety and efficacy, particularly in healthy populations and special groups (such as immunocompromised individuals and the elderly), which will help clarify the potential health benefits of Akk11 in different populations. Furthermore, long‐term follow‐up studies should be designed to assess the lasting effects of Akk11 on host health, particularly in terms of improving gut health, immune response, and potentially metabolic effects.

## 5 Conclusion

In conclusion, *Akkermansia muciniphila* Akk11 exhibits a favorable safety profile, supported by comprehensive genotypic, phenotypic, and toxicological evaluations. Administration of Akk11, containing mixed live and non‐viable cells, is well tolerated, with no observed toxicological effects in animal models. These findings provide a solid scientific basis for the future development and clinical application of Akk11 as a next‐generation probiotic.

## Author Contributions

X.W. was responsible for conceptualization, investigation, data curation, formal analysis, and drafting the initial manuscript. Y.F., Y.D., Y.Z., X.T. and Z.G. contributed to software, methodology, data curation, and revising the manuscript critically for important intellectual content. Y.S. and S.F. oversaw project administration, resource allocation, supervision, formal analysis, software, validation, funding acquisition and visualization.

## Ethics Statement

All animal experiments were conducted in strict accordance with the EU Directive 2010/63/EU on the protection of animals used for scientific purposes. The study protocol was reviewed and approved by the Institutional Animal Care and Use Committee (IACUC) of Shanghai Shengchang Biotechnology Co. Ltd. All procedures were designed and reported following the ARRIVE guidelines (https://arriveguidelines.org). Euthanasia was performed under anesthesia induced by carbon dioxide inhalation, followed by confirmatory cervical dislocation, as mandated by the EU Directive, ARRIVE guideline and IACUC protocols. Every effort was made to minimize animal suffering and reduce the number of animals used, in alignment with the 3R (Replacement, Reduction and Refinement) principles.

## Conflicts of Interest

Some of the authors are employees of the Wecare Probiotics Co. Ltd., which also funded this research. The authors declare that this affiliation did not influence the objectivity of the research. The funders had no role in the design of the study; in the collection, analyses, or interpretation of data; in the writing of the manuscript; or in the decision to publish the results.

## Supporting information


**Figure S1:** fsn371154‐sup‐0001‐FigureS1.tif.

**Table S1:** fsn371154‐sup‐0002‐TablesS1‐S3.xlsx.

## Data Availability

The data used in the current study is available upon reasonable request to the corresponding author. The data are not publicly available due to privacy restrictions.
