# Supplementary figures and images for: Strain‐Specific Safety Evaluation of Akkermansia muciniphila Akk11: Comprehensive Genotypic, Phenotypic, and Toxicological Assessment
Source: Food Sci Nutr. 2025 Nov 4;13(11):e71154. doi: 10.1002/fsn3.71154 (PMC12586882; doi:10.1002/fsn3.71154)

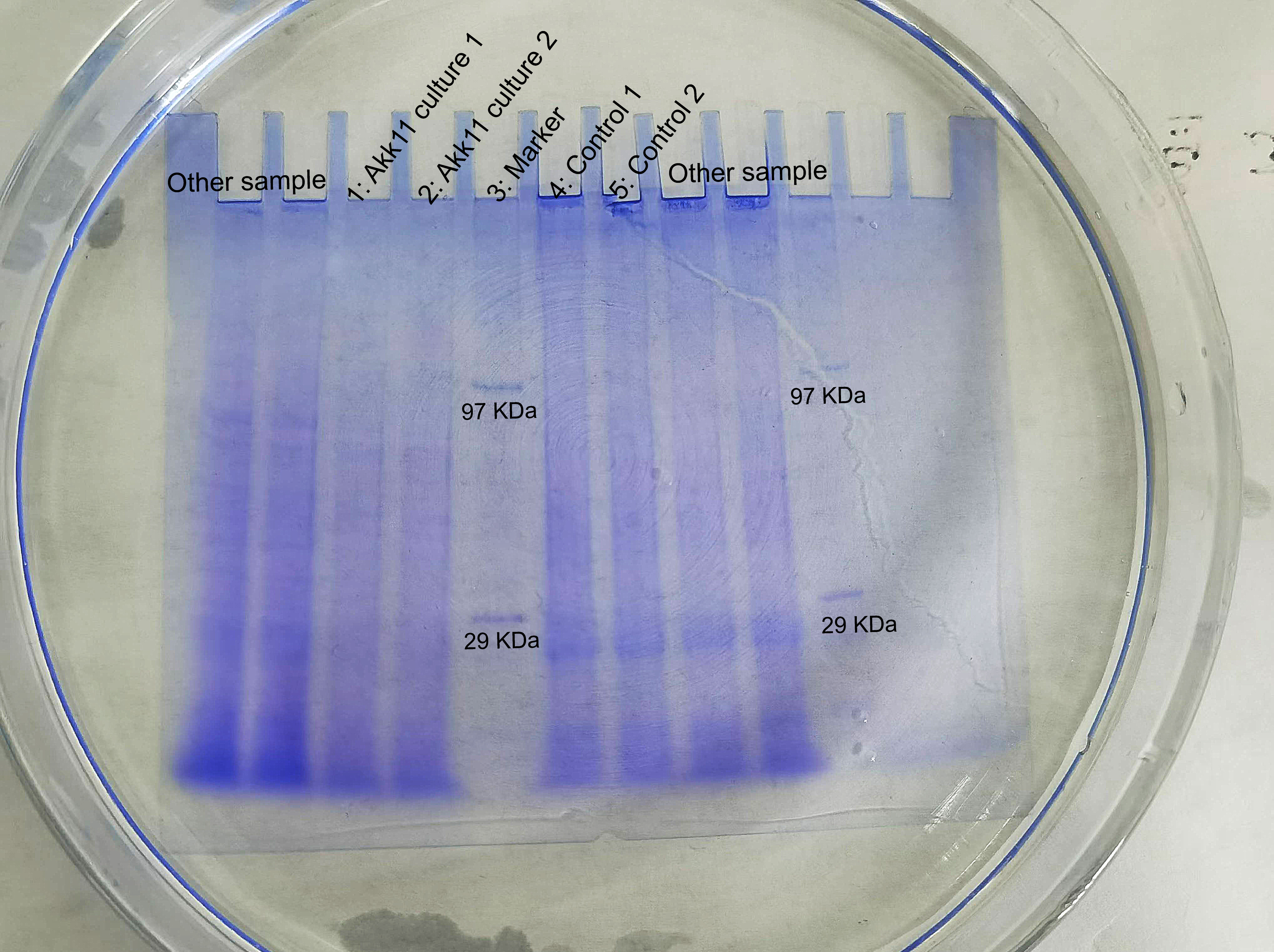

Supplement: Supplementary file 1 — Figure S1: fsn371154‐sup‐0001‐FigureS1.tif. [file FSN3-13-e71154-s002.tif]
